# Interaction Between *ACE* I/D and *ACTN*3 R557X Polymorphisms in Polish Competitive Swimmers

**DOI:** 10.2478/hukin-2014-0067

**Published:** 2014-10-10

**Authors:** Agata Grenda, Agata Leońska-Duniec, Mariusz Kaczmarczyk, Krzysztof Ficek, Paweł Król, Paweł Cięszczyk, Piotr Żmijewski

**Affiliations:** 1 Department of Physical Education and Sport, West Pomeranian Technological University, Szczecin, Poland.; 2 Faculty of Physical Culture and Health Promotion, University of Szczecin, Szczecin, Poland.; 3 Faculty of Tourism and Recreation, Academy of Physical Education and Sport, Gdansk, Poland.; 4 University of Rzeszow, Department of Physical Culture, Rzeszow, Poland.; 5 Institute of Sport in Warsaw, Poland.

**Keywords:** ACE, ACTN3, gene polymorphism, swimming

## Abstract

We hypothesized that the ACE ID / ACTN3 R577X genotype combination was associated with sprint and endurance performance. Therefore, the purpose of the present study was to determine the interaction between both ACE ID and ACTN3 R577X polymorphisms and sprint and endurance performance in swimmers. Genomic DNA was extracted from oral epithelial cells using GenElute Mammalian Genomic DNA Miniprep Kit (Sigma, Germany). All samples were genotyped using a real-time poly- merase chain reaction. The ACE I/D and the ACTN3 R577X genotype frequencies met Hardy-Weinberg expectations in both swimmers and controls. When the two swimmer groups, long distance swimmers (LDS) and short distance swimmers (SDS), were compared with control subjects in a single test, a significant association was found only for the ACE polymorphism, but not for ACTN3. Additionally, four ACE/ACTN3 combined genotypes (ID/RX, ID/XX, II/RX and II/XX) were statistically significant for the LDS versus Control comparison, but none for the SDS versus Control comparison. The ACE I/D and the ACTN3 R577X polymorphisms did not show any association with sprint swimming, taken individually or in combination. In spite of numerous previous reports of associations with athletic status or sprint performance in other sports, the ACTN3 R577X polymorphism, in contrast to ACE I/D, was not significantly associated with elite swimming status when considered individually. However, the combined analysis of the two loci suggests that the co-occurrence of the ACE I and ACTN3 X alleles may be beneficial to swimmers who compete in long distance races.

## Introduction

Elite athletic performance is a complex phenotype determined by several environmental factors such as diet, physical training, and sociocultural factors. Although all these factors are certainly acknowledged as key components contributing to sport performance, there remains a belief that there is a genetic component to the success of elite athletes. A recent study has identified in excess of 240 gene variants as potential genetic markers associated with fitness-related phenotypes, although few of these variants have been associated with elite-level athletic performance (Bray et al., 2009).

The most frequently investigated genetic markers in the context of athletic predisposition are the *ACE* and the *ACTN3* polymorphisms (Pokrywka et al., 2013). Variants in both these genes have been reported to be associated with elite athletic performance and with normal, quantitative physical performance traits in the general population (Bustamante-Ara et al., 2010; Pereira et al., 2013; Wang et al., 2013; Drozdovska et al., 2013).

The human angiotensin converting enzyme gene (*ACE*) is located on chromosome 17 in position 17q23.3 (Rieder et al., 1999). The product of this gene (an enzyme converting angiotensin I into II) is acknowledged to be a key-element in the renin angiotensin system (RAS), a system responsible for the regulation of blood pressure – one of the main factors determining the efficiency of the whole body. The most widely studied *ACE* polymorphism is the restriction fragment length polymorphism consisting of the presence (insertion, I) or absence (deletion, D) of a 287 base pair Alu repeat sequence in intron 16. In this case, three *ACE* genotypes include DD and II homozygotes and ID heterozygotes. The I allele is associated with lower ACE activity in both serum and tissue compared with the D allele (Rigat et al., 1990).

In reports regarding association between the *ACE* genotype and training, the DD genotype seems to impart an advantage in the development of short duration aerobic performance (Cam et al., 2007). Moreover, subjects with at least one D allele have shown greater strength gains and muscle volume after isometric strength training in quadriceps muscles (Charbonneau et al., 2008). Finally, several reports proved that the D allele was associated with elite power athlete status. On the other hand, an excess of the I allele has been associated with some aspects of endurance performance (Bray et al., 2009). The allele I and II genotypes are related to greater improvements in medium duration aerobic performance (Cam et al., 2007) as well as being associated with an increase in the endurance and effectiveness of muscles and are also responsible for an increase in the proportion of free fibers (type I muscle fibers) (Macarthur and North, 2005). Nevertheless, some studies do not confirm these observations (Orysiak et al., 2013).

The second most frequently investigated gene in the context of sport is *ACTN3*, coding the protein α-actinin-3. Alpha-actinins are an ancient family of actin binding proteins (Mills et al., 2001) that play structural and regulatory roles in cytoskeletal organization. In skeletal muscle, two alpha-actinin proteins (α-actinin-2 and α-actinin-3) comprise an important structural component of the Z disc, where they anchor actin thin filaments, helping to maintain the myofibrillar array. Besides their mechanical role, both sarcomeric alpha-actinins interact with proteins involved in numerous signalling and metabolic pathways (Mills et al., 2001). While α-actinin-2 is expressed in all types of muscle fibers, the expression of α-actinin-3 is almost exclusively restricted to fast, glycolytic type II fibers (Clarkson et al., 2005).

In 1999, North et al. (1999) identified a common polymorphism in *ACTN3* R577X (dbSNP rs1815739) that resulted in the absence of α-actinin-3 in more than one billion people worldwide. The first evidence that a mononucleotide difference in DNA sequence was associated with power ability referred to the R577X polymorphism of the *ACTN3* gene (Yang et al., 2003), where the translation (C > T) at nucleotide position 1747 in the *ACTN3* coding sequence converts an arginine (R) to a stop codon (X) at residue 577 (Mills et al., 2001). This variation creates two different versions of the *ACTN3* gene, both of which are common in the general population: the 577R allele is the normal, functional version of the gene, whereas the 577X allele contains a sequence change that completely prevents the production of functional α-actinin-3 protein (North et al., 1999).

The 577R allele and 577RR genotype of the *ACTN3* gene are associated with top-level, power-orientated athletic performance in a wide array of ethnic groups (Yang et al., 2003; Niemi et al., 2005; Cięszczyk et al., 2011). Additionally, there is a positive association between the presence of the R allele and the capacity to perform high-power muscle contractions (Clarkson et al., 2005). Furthermore, Vincent et al. (2007) showed that the surface area percentage and number of type IIx (fast-twitch glycolytic) fibers were greater in the RR than the XX genotype of young healthy men. Though the XX genotype is not associated with any known disease phenotype, an α-actinin-3-deficiency is believed to preclude top-level athletic performance in ‘pure’ power and sprint sports (North et al., 1999). On the other hand, Yang et al. (2003) hypothesized that a total deficiency of the α-actinin-3 protein may confer some beneficial effect on endurance performance. Some studies have reported that the loss of α-actinin-3 expression in a knockout mouse model results in a shift in fast muscle metabolism toward the more efficient aerobic pathway and an increase in intrinsic endurance performance (MacArthur and North, 2007). Additionally, Yang’s (2003) hypothesis seems to be supported by the fact that the XX *ACTN3* genotype occurs at higher frequency in some groups of elite endurance athletes (Niemi et al., 2005; Eynon et al., 2009b).

Taking these data all together, we hypothesized that the *ACE* ID / *ACTN3* R577X genotype combination was associated with sprint and endurance performance. Therefore, the purpose of the present study was to determine the interaction between both *ACE* ID and *ACTN3* R577X polymorphisms and sprint and endurance performance in swimmers.

## Material and Methods

### Ethics Committee

The Pomeranian Medical University Ethics Committee approved the study and written informed consent was obtained from each participant. The study complied with the guidelines set out in the Declaration of Helsinki and the ethics policy of the Szczecin University (Kruk, 2013).

### Subjects

Various methods were used to obtain the samples, including targeting national teams and providing information to national coaching staff and athletes attending training camps. After informed consent, 196 Polish swimmers (104 males and 92 females, 20.3 ± 2.7 years) were recruited for this study. All participants were Caucasians to minimise the influence of racial genetic skew and to remove any potential population stratification problems. The swimmers were divided into two homogeneous groups, based on their competitive distance and values of relative contribution of the aerobic or/anaerobic energy systems: long distance swimmers (LDS, n=49, 24 males, 25 females), more than 500 m (mainly aerobic events) and short distance swimmers (SDS, n=147, 80 males, 67 females), between 50 and 200 m (mainly anaerobic events). All investigated swimmers had been finalists of the Polish National Championships. Additionally, 7 of them had participated in the Olympic Games and 48 of them had taken part in the World Championships or European Championships. The whole group of swimmers included 8 World Championship medalists, 15 European Championship medalists and 128 Polish Championship medalists. A control group of healthy individuals (*n* = 379, 222 males and 157 females, 22.6 ± 2.8 years) was also selected from the Polish population (college students) with no background in swimming.

### Genotyping

The buccal cells donated by the subjects were collected in Resuspension Solution (Sigma, Germany) with use of Sterile Foam Tipped Applicators (Puritan, USA). DNA was extracted from the buccal cells using GenElute Mammalian Genomic DNA Miniprep Kit (Sigma, Germany) according to the producer protocol.

### ACE genotyping

PCR amplification of the polymorphic region of the ACE gene containing either the insertion (I) or dele-tion (D) fragment was performed. Only one pair of primers (ACEfor: CTG GAG ACC ACT CCC ATC CTT TCT and ACErev: GAT GTG GCC ATC ACA TTC GTC AGA) was used to determine the ACE genotype, yielding amplification products of approximately 490 bp (for I allele) and 190 bp (for D allele). The 10 μl PCR consisted of: 1 μl DNA isolate; 0.5 U DNA recombinant Taq polymerase in buffer (pH = 8.0; Sigma, Germany); 1x PCR buffer (pH = 8.7; Sigma, Germany); 1.5 mM MgCl 2; 4 pM primer ACEfor and ACErev (Oligo, Poland) in TE buffer (pH = 8); 0.75 nM of each dNTP. The thermal-time PCR was as follows: initial denaturation at 94^°^C for 300 s, 30 cycles (denaturation at 92^°^C for 60 s, primer annealing at 58^°^C for 60 s, chain extension at 72^°^C for 150 s) and final extension at 72^°^C for 360 s. The reaction was performed in two samples per isolate. Amplification products were visualized in UV light by using 1.5% agarose gels stained with ethidium bromide.

### ACTN3 genotyping

The 290 bp fragment of exon 15 of the *ACTN3* gene was amplified by PCR using the forward primer : 5′CTGTTGCCTGTGGTAAGTGGG-3′ and the reverse primer : 5′TGGTCACAGTATGCAGGAGGG-3′ as recommended by Mills et al. (2001). PCR reaction mix (total volume 10 μl) contained 1.5 mM MgCl2, 0.75 nM of each deoxynucleoside triphosphate (Novazym, Poland), 4 pM of each primer (Genomed, Poland), 0.5 U of Taq DNA polymerase (Sigma, Germany), and 1 μl (30–50 ng) of template DNA. After a first step consisting of 95°C for 5 min, 35 cycles of amplification were performed by using denaturation at 95°C for 30 s, annealing at 60°C for 30 s, and elongation at 72°C for 30 s and a final cycle at 72°C for 10 min (Eynon et al., 2009). The amplified PCR fragments were subsequently digested with *Dde I* endonuclease (Fermentas, Lithuania) in a condition recommended by the supplier (Mills et al., 2001). The alleles 577R and 577X were distinguished by the presence (577X) or absence (577R) of a *Dde I* restriction site. Digestion of PCR products of the 577X allele yields bands of 108, 97 and 86 bp, whereas digestion of PCR products of the 577R allele yields bands of 205 and 86 bp. The digested products were separated by 3% agarose gel electrophoresis, stained with ethidium bromide, and visualized in UV light.

### Statistical analysis

The Hardy-Weinberg equilibrium (HWE) for *ACE* and *ACTN3* genotypes was assessed separately in swimmers and control subjects with a χ^2^ test. The associations between *ACE* I/D and *ACTN3* R577X genotypes (individually and in combination) and swimmers’ status were examined using a multinomial logistic regression with a three-category outcome variable: LDS, SDS and control. The analysis of the individual effects of these variants was based on three genetic models: general (II vs ID vs DD, XX vs RX vs RR), dominant (II+ID vs DD, XX+RX vs RR) and recessive (II vs ID+DD, XX vs RX+RR). In the combined analysis, all combinations of *ACE* and *ACTN3* genotypes were determined under a general model for each variant. Next, *ACE* and *ACTN3* dominant model genotype combinations (II+ID/XX+RX, II+ID/RR, DD/XX+RX, DD/RR) as well as *ACE* and *ACTN3* recessive model genotype combinations (II/XX, II/RX+RR, ID+DD/XX, ID+DD/RX+RR) were examined. For individual and combined analysis, two types of significance tests were conducted. First, two swimmer groups were compared with control subjects in a single test – likelihood ratio (LR) p value. Then, each swimmer group (LDS and SDS) was compared with control subjects (Wald statistics based p value). The odds ratio (OR) with 95% confidence intervals were calculated. All calculations were done in R (version 2.15.2, http://cran.r-project.org) using three packages: genetics, nnet, car and effects. The p value of less than 0.05 was considered significant.

## Results

The *ACE* I/D and the *ACTN3* R577X genotype frequencies (Table 1) met Hardy-Weinberg expectations in both swimmer groups (p=0.294 and p=0.337 for *ACE* I/D and *ACTN3* R577X, respectively) and controls (p=0.920 and p=0.374 for *ACE* I/D and *ACTN3* R577X, respectively).

When the two swimmer groups, long distance swimmers (LDS) and short distance swimmers (SDS), were compared with control subjects in a single test, a significant association was found only for the *ACE* polymorphism (LR Chi-square 22.58 df=4, p=0.0002), but not for *ACTN3* (LR Chi-square 5.60, df=4, p=0.231). Assuming a general model, relative to control subjects, carriers of the ID genotype were more likely to be found in the LDS group than DD homozygotes (8.5% vs 1.9%, OR 5.14 [1.52–17.41], p=0.009) as were the II homozygotes (16.7% vs 1.9%, OR 10.83 [3.12–37.57], p=0.0002). The odds ratios for a dominant (II+ID versus DD) and recessive (II versus ID+DD) model were 6.76 (2.06–22.17), p=0.002 and 3.04 (1.64–5.65), p=0.0004, respectively. There were no significant *ACE* genotype-dependent differences in the likelihood of being classified as a short distance swimmer under general, dominant and recessive models. Likewise, no significant differences were observed for *ACTN3*, either between LDS and control subjects or SDS and control subjects.

**Figure 1:**
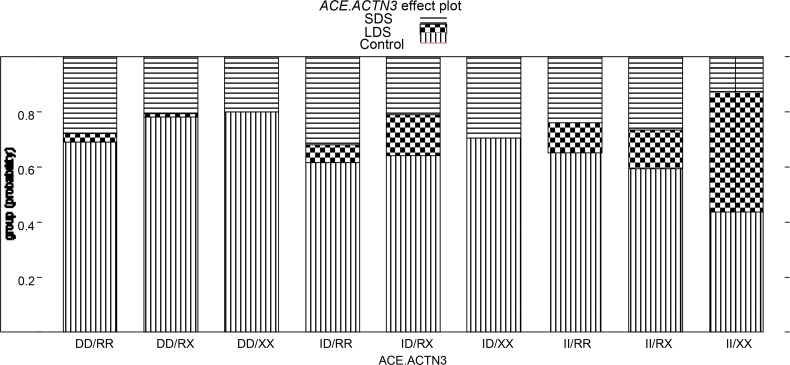
***ACE/ACTN****3 combined genotype-dependent probability of being categorized to long distance or short distance swimmers*

Next, genotype combinations of *ACE* and *ACTN3* were determined and they were compared between combined swimmer groups (LDS and SDS) and control subjects in a single test (LR Chi-square 50.6, df=16, p<0.0001, Table 2). All possible 9 *ACE*/*ACTN3* genotype combinations were present in at least one group. Four *ACE*/*ACTN3* combined genotypes (ID/RX, ID/XX, II/RX and II/XX) were statistically significant for the LDS versus Control comparison, but none for the SDS versus Control comparison (Figure 1). With respect to control individuals, the carriers of ID/RX (14.9%, OR 5.48 [1.21–24.80], p=0.027), II/RX (14.1%, OR 5.59 [1.13–27.34], p=0.034) and II/XX (43.8%, OR 23.51 [4.04–136.80], p=0.0004) were more likely to be found in the LDS group than DD/RR homozygotes (2.9%). In contrast, carriers of the ID/XX combined genotype were not present in the LDS group (0% vs 2.9%, p<0.0001). No significant differences in the frequencies of combined genotypes were observed between SDS and control subjects.

Additionally, the combined analysis was conducted for dominant (*ACE*: II+ID vs DD, *ACTN3*: XX+RX vs RR) and recessive (*ACE*: II vs ID+DD, *ACTN3*: XX vs RX+RR) genetic models (Table 3). The frequency of combined genotypes for dominant and recessive models differed significantly in a single test comparing LDS and SDS groups with the control subjects (LR Chi-square 23.8, df=6, p=0.0006; LR Chi-square 29.1, df=6, p=0.00006, for dominant and recessive models, respectively). For dominant *ACE* and *ACTN3* models, with respect to control individuals, the carriers of at least one *ACE I* allele and at least one *ACTN3* X allele (II/XX, II/RX, ID/XX, ID/RX) were significantly more frequent in the LDS group compared with the carriers of two homozygous DD and RR genotypes (13.5% vs 2.9%, OR 5.04 [1.16–21.80], p=0.030). For *ACE* and *ACTN3* recessive models, the carriers of two homozygous genotypes (II/XX) were also over-represented in the LDS group compared with carriers of at least *ACE* D and at least one *ACTN3* R allele (43.8% vs 7.3%, OR 9.14 [2.99–27.96], p=0.0001, Table 3). No differences were observed between the SDS and control group.

## Discussion

Interaction between genes has long been appreciated to be important in understanding the function of genetic pathways (Phillips, 2008). In the present study we examined two common genetic variants: *ACE* I/D and *ACTN3* R577X polymorphisms in adult elite swimmers of Caucasian origin, individually as well as in combination using the complex *ACE*/*ACTN3* genotypes, with regard to swimmers’ competitive racing distances.

There are two major findings of the present study. The first is the over-representation of the *ACE* I allele (under general, *ACE* I allele dominant and recessive models) in long distance swimmers compared to control individuals. Our observation is similar to that of previous studies investigating the *ACE* I/D polymorphism in elite swimmers that demonstrated an excess of the I allele among middle or long distance elite swimmers (Nazarov et al., 2001; Tsianos et al., 2004). However, the lack of association of the *ACE* I/D polymorphism with elite short distance swimming is unexpected in the light of previous observations (Nazarov et al., 2001; Costa et al., 2009; Woods et al., 2001). A possible explanation could be the sampling bias affecting the alleles of the *ACE* I/D polymorphism among swimmers and the control group. It is, however, unlikely, at least regarding the control group, as the *ACE* D allele frequency (0.55, data not shown) in healthy individuals without a background in swimming in our study was similar to that reported by Nazarov et al. (2001) (0.50). It must also be noted that the studies examining the *ACE* I/D variant in elite swimmers have provided conflicting results. Wang et al. (2013) demonstrated that either the *ACE* D allele (Caucasians) or the *ACE* I allele (East Asians) were over-represented in short distance swimmers depending on the population studied. The previous observation puts in question the causative effect of the *ACE* I/D polymorphism on physical performance in general and raises the possibility that alleles of the *ACE* I/D polymorphism are merely the marker proxies of other potential causal alleles, creating different, ethnicity-dependent haplotypes.

The second observation from our study was that although the *ACTN3* R577X polymorphism was not associated with a predisposition to short or long distance swimming when examined alone, when using a combined analysis strategy, a gene-gene interaction between *ACTN3* R577X and *ACE* I/D was demonstrated. At first, we examined all possible *ACTN3* R577X and *ACE* I/D combined genotypes (Table 2, Figure 1). Next, we conducted comparisons for the *ACE*/*ACTN3* genotype combinations with the assumption that the *ACE* I/D and *ACTN3* R577X polymorphisms followed the same inheritance pattern, dominant or recessive (Table 3). Both approaches provided evidence for an interaction between these two loci. Four combined *ACE*/*ACTN3* genotypes: ID/RX, ID/XX, II/RX and II/XX were associated with a predisposition to long distance swimming. With one exception (combined genotype ID/XX), the chance of being a long-distance swimmer rather than in a control group was greater (from 5.48 to 23.51) among carriers of these combined genotypes compared with the reference DD/RR genotype, an optimum power muscularity-oriented genotype (Pereira et al., 2013). Surprisingly, a combination of *ACE* ID and homozygous *ACTN3* XX genotype was not present in the long distance swimmers, although 10.2% of short distance swimmers (29.4% of all ID/XX) and 9.5% of controls (70.6% of all ID/XX) had this genotype (Figure 3). That resulted in a very low value of the odds ratio (range of 10^−9^) contrasting markedly with odds associated with ID/RX, II/RX and II/XX combined genotypes. Further, the analysis of gene-gene interaction using dominant and recessive models showed that the carriers of the *ACE* I allele were more likely to be classified as long distance swimmers when the 577X allele was also present along with the I allele. In contrast, the carriers of at least one I allele (II, II+ID) were not over-represented in the LDS group if they possessed at least one R allele as well (II+ID/RR, II/RR+RX). Thus, surprisingly, although the *ACTN3* genotypes did not differ significantly between the LDS and control group, the 577R allele seemed to counteract the effect of the I allele.

Although the *ACE* I allele has been reported to be associated with endurance sports or endurance performance (Bray et al., 2009), including a predisposition to long distance swimming (Nazarov et al., 2001; Tsianos et al., 2004), the lack of association between long or short distance swimming and the *ACTN3* R577X variant may seem unexpected. Previous studies demonstrated the association of the 577X or the XX genotype with elite endurance athletic status (Niemi et al., 2005; Eynon et al., 2009) as well as the 577R or the RR genotype with elite, power-oriented athletic status (Yang et al., 2003; Eynon et al., 2009b).

Given the relevance of strength and power in short distance events (Costa et al., 2009), and the fact that strength and speed are major determinants of a sprint swimmers’ performance (Toussaint and Vervoorn, 1999), we expected the 577R allele to be over-represented in a group of swimmers who specialized in short distance races. However, studies reporting the association of the 577R or the RR genotype with elite, power-oriented athletic status included athletes engaged in various sport disciplines (sprinters, throwers, jumpers, endurance road cyclists, marathon runners or triathletes) with only a minor representation of swimmers. For instance, short distance swimmers accounted for only about 2–3,9% of all examined power-oriented athletes (Yang et al., 2003) or did not include swimmers at all (Niemi et al., 2005; Eynon et al., 2009).

Recently, two studies have been conducted on a group of swimmers exclusively and, similarly to our study, showed no association between the *ACTN3* R577X polymorphism and elite swimming status (Wang et al., 2013; Ruiz et al., 2013). Ruiz et al. (2013) found no over- or under-representation of any *ACTN3* R577X genotypes in 88 Spanish Caucasian swimmers compared with non-athletic subjects. Wang et al. (2013) examined the *ACE* I/D and *ACTN3* R577X polymorphisms in elite Caucasian and East Asian (Japanese and Taiwanese) swimmers, but not the interaction between the two loci. To the best of our knowledge, the present study is the first to examine the association between the *ACE* I/D and the *ACTN3* R577X polymorphisms, independently and in combination, among competitive swimmers.

To date, the combined effect of these two common genetic variants have been examined in relation with athletic status in sprinters (Eynon et al., 2009a) and wrestlers (Kikuchi et al., 2012) as well as with muscle performance or exercise-related phenotypes both in athletes (Ginevičien et al., 2011) and non-athletic populations (Bustamante-Ara et al., 2010; Pereira et al., 2013). Nonetheless, these studies have provided largely inconsistent results. The *ACTN3* RR genotype with the *ACE* I allele and the *ACE* II genotype with the *ACTN3* R allele were found to be over-represented in the group of Israeli sprinters (Eynon et al., 2009a), while in a Lithuanian population grip strength and vertical jump performance were better in the athletes with the *ACE* II and *ACTN3* XX genotypes.

In contrast to these studies, the 577R allele and *ACE* DD genotype were found to modulate muscle phenotypes in response to high-speed power training in older women (Pereira et al., 2013). On the other hand, no associations were found between *ACE* DD and *ACTN3* RR + RX genotype combination and jumping and sprint ability in young healthy adults and upper and lower body muscular strength in nonagenarian women (Bustamante-Ara et al., 2010). Further studies analyzing swimming performance and selected gene polymorphisms (Grenda et al., 2014) are need.

The strength of our study lies in a comprehensive analysis of the *ACE*/*ACTN3* genotype combinations. Unlike in other studies, instead of arbitrary, literature-based selection of sport-related optimum genotypes combinations, we examined the gene-gene interaction under dominant and recessive models, thus we were able to ascertain effects of combined genotypes that might have been hidden otherwise. The sample size is comparable with those studies conducted among swimmers (Wang et al., 2013; Ruiz et al., 2013).

## Practical applications

Identifying genetic polymorphisms which enhanced sport performance is helpful in understanding individual variations in health-and exercise-related phenotypes. In case of the presented article, the main assumption is the interaction between *ACE* I/D and *ACTN3* R557X polymorphisms, which might benefit sport performance in swimming. This kind of information (i.e., information concerning the polymorphisms related to athletic performance) may be used to develop genetic tests that identify athletic talent and aid in preventing injury. Furthermore, it is likely that in the near future, knowledge about specific genetic markers will allow for specific steering of sport training programs and determining the probable extent of adaptive response to implemented training.

## Conclusions

In conclusion, *ACE* I/D and *ACTN3* R577X polymorphisms did not show any association with performance in sport swimming, taken individually or in combination. In spite of numerous previous reports on associations with athletic status or sprint performance in other sports, the *ACTN3* R577X polymorphism, in contrast to *ACE* I/D, was not significantly associated with elite swimming status when considered individually. However, the combined analysis of the two loci suggests that the cooccurrence of the *ACE* I and *ACTN3* X alleles may be beneficial to swimmers who compete in long distance races.

## Figures and Tables

**Table 1:** **ACE** and **ACTN**3 genotype distributions among swimmers and control subjects

Group	*ACE*			*ACTN3*		

	DD^*^	ID	II	RR^*^	RX	XX
LDS (n=49)	3 (1.9)	25 (8.5)^†^	21 (16.7)^†^	15 (6.2)	27 (10.8)	7 (8.5)
SDS (n=147)	37 (23.7)	80 (27.3)	30 (23.8)	71 (29.3)	56 (22.3)	20 (24.4)
Control (n=379)	116 (74.4)	188 (64.2)	75(59.5)	156 (64.5)	168 (66.9)	55 (67.1)

***ACE****: LR Chi-square 22.58 df=4, p=0.0002;* ***ACTN****3: LR Chi-square 5.60, df=4, p=0.231); LDS – long distance swimmers; SDS – short distance swimmers;*

* reference genotype for comparisons;

† significant when compared with reference genotype relative to the control group

**Table 2:** Combined **ACE** I/D and **ACTN**3 R577X genotype frequencies among swimmers and control subjects

Group	II			ID			DD		

	RR	RX	XX	RR	RX	XX	RR^*^	RX	XX
LDS (n=49)	5 (10.9)	9 (14.1) ^†^	7 (43.8)^†^	8 (6.3)	17 (14.9)^†^	0 (0)^†^	2 (2.9)	1 (1.4)	0 (0)
SDS (n=147)	11 (23.9)	17 (26.6)	2 (12.5)	41 (32.0)	24 (21.1)	15 (29.4)	19 (27.9)	15 (20.6)	3 (20.0)
Control (n=379)	30 (65.2)	38 (59.4)	7 (43.8)	79 (61.7)	73 (64.0)	36 (70.6)	47 (69.1)	57 (78.1)	12 (80.0)

LR Chi-square 50.6, df=16, p<0.0001; LDS – long distance swimmers, SDS – short distance swimmers;

* reference genotype (DD/RR) for comparisons;

† significant when compared with reference genotype relative to the control group

**Table 3:** Combined genotype frequencies among swimmers and control subjects assuming ACE/ACTN3 dominant models and **ACE/ACTN**3 recessive models

Group	*ACE* and *ACTN3* dominant models combinations				*ACE* and *ACTN3* recessive models combinations			

	DD/RR^*^	DD/XX+RX	II+ID/RR	II+ID/XX+RX	ID+DD/RR+RX^*^	ID+DD/XX	II/RR+RX	II/XX
LDS (n=49)	2 (2.9)	1 (1.1)	13 (7.5)	33 (13.5)^†^	28 (7.3)	0 (0)	14 (12.7)	7 (43.8)^†^
SDS (n=147)	19 (27.9)	18 (20.5)	52 (29.9)	58 (23.7)	99 (25.9)	18 (27.3)	28 (25.5)	2 (12.5)
Control (n=379)	47 (69.1)	69 (78.4)	109 (62.6)	154 (62.9)	256 (66.8)	48 (72.7)	68 (61.8)	7 (43.8)

Dominant models – LR Chi-square 23.8, df=6, p=0.0006; Recessive models – LR Chi-square 29.1, df=6, p=0.00006, LDS – long distance swimmers, SDS – short distance swimmers;

* model-specific reference genotype for comparisons;

† significant when compared with reference genotype relative to the control group
